# Hypertrichosis Cubiti Presenting in a Female Child: A Case Report

**DOI:** 10.7759/cureus.83406

**Published:** 2025-05-03

**Authors:** Matthew K Mlachak, Michelle A Jahnke

**Affiliations:** 1 Research, The Ohio State University, Columbus, USA; 2 Dermatology, New Horizon Dermatology, Inc., Ravenna, USA

**Keywords:** hair disorder, hypertrichosis, hypertrichosis cubiti, pediatric dermatology, skin disorder

## Abstract

Hypertrichosis cubiti is a rare form of unusual hair growth in the arms around the elbow area. Here, we report a case of hypertrichosis cubiti in a six-year-old female child with a sacral cleft, otherwise of normal health. Specifically, terminal hair growth was observed in this case. The excessive hair growth often poses aesthetic concerns for patients and can be treated to avoid emotional distress regarding physical appearance. Management of the condition typically involves common hair removal techniques, and over-the-counter depilatory creams and shaving were used to treat this case.

## Introduction

Hypertrichosis cubiti, commonly known as “hairy elbow syndrome”, is a highly underreported condition in biomedical literature. It can be identified through the appearance of excessive hair growth on the lateral aspects of the lower third of the upper arm and the upper third of the forearm. The first reported case was identified in 1970 by Beighton in a young female whose maximum hair growth was noted at the age of five, with the condition becoming less visible as she aged [1]. Hypertrichosis cubiti has previously been identified to accompany genetic conditions such as Wiedemann-Steiner syndrome [2], although the condition has been seen to manifest independently [3]. Here, we present the infrequent condition, hypertrichosis cubiti, in a six-year-old female child. 

## Case presentation

**Figure 1:**
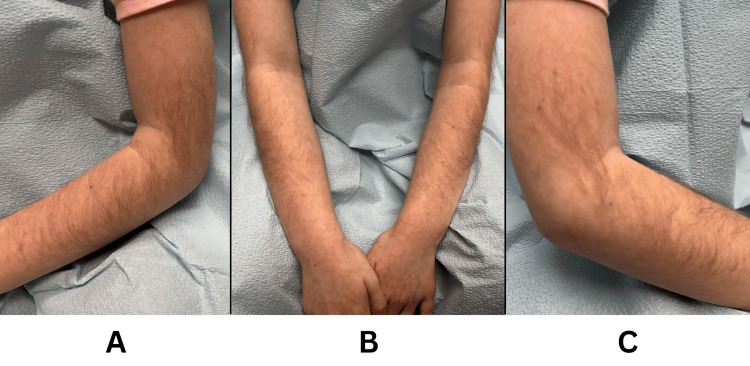
Hypertrichosis cubiti on the extensor surfaces of the regions between the forearms and upper arms (A) left arm, (B) bilateral, (C) right arm

A six-year-old female child presented with long terminal hair growth on the extensor surfaces of the regions starting at her forearms and through the upper arms, with the highest concentration of hair growth around the elbow (Figures 1A, 1C). The growth was bilateral and symmetric (Figure 1B). Upon examination, no other abnormal hair growth was observed in the upper extremities of the female. The hair growth was first noticed by the mother at the age of three, with growth progressing over time. The mother reported the patient pulling out the hair at a younger age, with the hair growing back rapidly. No family history of hypertrichosis was reported. Interestingly, a sacral cleft was discovered, but no other physical or mental abnormalities were observed in the female. Furthermore, the patient did not appear to be of short stature and was identified in a normal percentile of growth. The main concern of the patient was the removal of the hair due to its abnormal quantity. Additionally, the patient and mother voiced their preference for the use of temporary removal options. Thus, the recommended treatment for the patient was the use of shaving and over-the-counter chemical hair removal creams containing potassium thioglycolate and calcium hydroxide when hair growth is noticed. For safety purposes, the mother was instructed to apply the cream on the patient due to her young age. No complications or skin sensitivities have been reported thus far with treatment.

## Discussion

Hypertrichosis cubiti is an underreported dermatological condition that is identified by terminal or vellus hair growth on the extensor surface of the bottom portion of the upper arm and the upper portion of the forearm. The condition may present with other symptoms such as short stature and facial abnormalities, often due to underlying conditions such as Wiedemann-Steiner syndrome, or by itself [2-6]. In most reported cases, hair growth peaks within childhood, then progressively thins towards the end of adolescence. In the case presented, a similar hair growth progression was reported at the time of examination, although no hair growth regression has been observed thus far, most likely due to the young age of the patient.

The excessive hair growth characterized by hypertrichosis cubiti itself does not pose a threat to the overall health of a patient. In fact, its presence alone can be classified as a cosmetic issue of interest, granted it does not present as a part of an underlying condition [6]. Treatment options for the condition mainly consist of hair removal procedures such as shaving, trimming, waxing, laser removal, and, in some cases, electrolysis [7]. If depilatory creams are selected as a treatment, application instructions provided with the product should be followed closely, and application should be supervised by an adult. It is important to note that hair color and texture may pose a limit on the treatment method used.

In the current case, the patient with hypertrichosis cubiti also presented with a sacral cleft. Interestingly, in Wiedemann-Steiner syndrome, in which hypertrichosis cubiti is a common symptom, between one-quarter and one-third of patients also report a sacral cleft [8]. Since the patient in the current report did not present with any other common symptoms of Wiedemann-Steiner syndrome and no family history of the condition was reported, genetic testing for the condition was not explored. Nevertheless, Pavone and others reported a case of hypertrichosis cubiti in a patient without Wiedemann-Steiner syndrome in which vertebral abnormalities were discovered [9]. Thus, an association between bone anomalies and hypertrichosis cubiti should be explored in patients without underlying genetic conditions. Without any immediate health concerns, the patient in this report will continue temporary hair removal treatments for hair growth management, with the hope that hair growth will regress towards the end of adolescence. 

## Conclusions

A six-year-old female child presented with hypertrichosis cubiti in a bilateral, symmetric form. No excessive hair growth was noted anywhere else on the patient. The female presented with a sacral cleft, but no other abnormalities were reported, and normal stature was observed. The main concern of the patient was the appearance of excessive hair growth, and temporary hair removal treatment options such as shaving and chemical hair removal were recommended.
